# The SUSTAIN Project: A European Study on Improving Integrated Care for Older People Living at Home

**DOI:** 10.5334/ijic.3090

**Published:** 2018-01-16

**Authors:** Simone R. de Bruin, Annerieke Stoop, Jenny Billings, Kai Leichsenring, Georg Ruppe, Nhu Tram, María Gabriela Barbaglia, Eliva A. Ambugo, Nick Zonneveld, Gerli Paat-Ahi, Henrik Hoffmann, Usman Khan, Viktoria Stein, Gerald Wistow, Manon Lette, Aaltje P.D. Jansen, Giel Nijpels, Caroline A. Baan, on behalf of the SUSTAIN consortium

**Affiliations:** 1 Centre for Nutrition, Prevention and Health Services, National Institute for Public Health and the Environment, Bilthoven, NL; 2 Department of General Practice and Elderly Care Medicine, Amsterdam Public Health research institute, VU University Medical Center, Amsterdam, NL; 3 Tranzo, Tilburg University, Tilburg, NL; 4 Centre for Health Service Studies, University of Kent, Canterbury, UK; 5 Austrian Interdisciplinary Platform on Ageing, Vienna, AT; 6 AGE Platform Europe, Brussels, BE; 7 Agency for Health Quality and Assessment of Catalonia, Barcelona, ES; 8 Department of Health Management and Health Economics, Institute of Health and Society, University of Oslo, Oslo, NO; 9 Vilans, National Center of Excellence in Long-term Care, Utrecht, NL; 10 Praxis Centre for Policy Studies, health policy program, Tallinn, EE; 11 Stiftung Gesundheit, Hamburg, DE; 12 European Health Management Association, Brussels, BE; 13 International Foundation for Integrated Care, Oxford, UK; 14 Personal Social Services Research Unit, Department of Social Policy, London School of Economics and Political Science, London, UK

**Keywords:** older people, integrated care, long-term care, implementation science, mixed methods, knowledge translation, European research

## Abstract

**Introduction:**

Integrated care programmes are increasingly being put in place to provide care to older people who live at home. Knowledge of how to further develop integrated care and how to transfer successful initiatives to other contexts is still limited. Therefore, a cross-European research project, called Sustainable Tailored Integrated Care for Older People in Europe (SUSTAIN), has been initiated with a twofold objective: 1. to collaborate with local stakeholders to support and monitor improvements to established integrated care initiatives for older people with multiple health and social care needs. Improvements focus on person-centredness, prevention orientation, safety and efficiency; 2. to make these improvements applicable and adaptable to other health and social care systems, and regions in Europe. This paper presents the overall structure and approach of the SUSTAIN project.

**Methods:**

SUSTAIN uses a multiple embedded case study design. In three phases, SUSTAIN partners: (i) conduct interviews and workshops with stakeholders from fourteen established integrated care initiatives to understand where they would prefer improvements to existing ways of working; (ii) collaborate with local stakeholders to support the design and implementation of improvement plans, evaluate implementation progress and outcomes per initiative, and carry out overarching analyses to compare the different initiatives, and; (iii) translate knowledge and experience to an online roadmap.

**Discussion:**

SUSTAIN aims to generate evidence on how to improve integrated care, and apply and transfer the knowledge gained to other health and social care systems, and regions. Lessons learned will be brought together in practical tools to inform and support policy-makers and decision-makers, as well as other stakeholders involved in integrated care, to manage and improve care for older people living at home.

## Introduction

Health and social care systems face the challenge of offering care and support to an increasing number of older people living at home [1]. This increase is partly due to an ageing population, but also governments in and outside Europe are pursuing agendas that seek to enable older people to participate in society and to live at home for as long as possible [2,3,4]. While a large proportion of older people are able to stay in their own homes, the prevalence of frailty, multimorbidity and disability increases with age, leading to a growing number of older people living in the community with multiple health and social care needs. These multiple needs may restrict social participation, and lead to reduced self-reliance and increased care dependence, which in turn may result in a higher utilisation of long-term care and support services [5,6,7,8].

Sustainable health and social care systems will need to be able to optimally support older people by addressing both their health and social care needs, while at the same time minimise service utilisation and expenditure. Integrated care is widely acknowledged to be a promising approach for meeting such challenges [9,10,11,12,13,14,15]. There are several definitions for the term ‘integrated care’ in place [16,17,18]. In this study, integrated care is defined as those initiatives that proactively seek to structure and coordinate care for older people in their own home environments, centred around their needs [12,13,14,19,20,21,22].

Numerous integrated care initiatives have been rolled out, in a wide range of settings and contexts, in and outside Europe [23,24,25,26], offering a rich and varied field of practical examples. Evaluations of these initiatives have established potential benefits of greater levels of service integration, but they have also highlighted limitations. For instance, evidence for the effectiveness of integrated care programmes for older people living at home remains inconsistent [14,27]. Also, knowledge of how to successfully implement and improve integrated care is still limited, as is knowledge of how to transfer these experiences to other contexts [28]. Furthermore, improvements to the current way of working in existing initiatives are considered necessary, to make them more person-centred, prevention-oriented, safe and efficient [24,27,29,30,31]. In addition, more insight into how to measure and evaluate (improvements in) integrated care programmes is needed to be able to capture outcomes and processes adequately and consistently across different programmes and evaluation studies.

### The SUSTAIN research project

To take a step forward in the development of integrated care, the cross-European research project called ‘SUSTAIN’ has been initiated, which stands for ‘Sustainable Tailored Integrated Care for Older People in Europe’ (http://www.sustain-eu.org). The project is funded under Horizon 2020 – the Framework Programme for Research and Innovation (2014–2020) from the European Commission (EC). SUSTAIN’s objectives are twofold: 1. to support and monitor improvements to established integrated care initiatives for older people living at home with multiple health and social care needs, and in so doing move towards more person-centred, prevention-oriented, safe and efficient care; and 2. to contribute to the adoption and application of these improvements to other health and social care systems, and regions in Europe. SUSTAIN is a four-year research project (2015–2019) carried out by thirteen partners from nine European countries: Austria (n = 1 partner), Belgium (n = 1 partner), Estonia (n = 1 partner), Germany (n = 1 partner), Ireland (n = 1 partner), Norway (n = 1 partner), Spain (n = 1 partner), the Netherlands (n = 4 partners) and the United Kingdom (n = 2 partners). The project team consists of dissemination partners focusing on knowledge translation and dissemination, and research partners who focus on supporting and evaluating improvements to the integrated care initiatives. The dissemination partners are from Belgium, Ireland, the Netherlands and the United Kingdom, and the research partners are from Austria, Estonia, Germany, Norway, Spain, the Netherlands and the United Kingdom. The aim of this paper is to describe SUSTAIN’s overall structure and intended approach and activities to generate evidence on improving integrated care and to transfer obtained knowledge to other health and social care systems, and regions.

## Methods

### Design and setting

Using a multiple embedded case study design [32,33], data are being collected from fourteen established integrated care initiatives for older people in seven European countries; Austria, Estonia, Germany, Norway, Spain, the Netherlands and the United Kingdom. The initiatives were already operating within their local health and social care systems. Each initiative, also referred to as ‘site’, is treated as one case study in the research. The project focuses on older people because complexity of needs and, consequently, care delivery tend to increase with age with the result that a more coordinated approach to service delivery, as pursued in the project, is required. Prior to the start of the project, SUSTAIN research partners invited integrated care initiatives in their networks within their countries, that were motivated to improve their current practice, to participate in the SUSTAIN project. Most sites already had a longstanding partnership with one of the SUSTAIN research partners. Criteria for including them in the study were defined by SUSTAIN research partners and drawn from the principles of the Chronic Care Model and related models [12,14,20,22,28,34]. Accordingly, initiatives should:

- Be willing and committed to improve their current practice by working towards more person-centred, prevention-oriented, safe and efficient care, which, in line with the EC’s stipulations, are SUSTAIN’s four key domains (see Table 1 for definitions);
- Focus on people aged 65 years and older, who live in their own homes and who have multiple health and social care needs;
- Support people to stay in their own homes (or local environments) for as long as possible;
- Address older people’s multiple needs, in other words, they should not be single disease oriented;
- Involve professionals from multiple health and social care disciplines working in multidisciplinary teams (e.g. nurses, social workers, pharmacists, dieticians, general practitioners);
- Be established, i.e. preferably operational for at least two years;
- Cover one geographical area or local site;
- Be mandated by one organisation that represents the initiative and that facilitates collaboration with SUSTAIN research partners.

**Table 1:** Definitions of SUSTAIN’s key domains.

**Person-centredness**	Involve older people and their informal carers in decision-making and planning their care process in order to tailor the delivery of care and support as much as possible to individual needs, preferences and capabilities, taking into account socio-demographic factors, cultural backgrounds and gender [35,36].
**Prevention orientation**	Preserve and promote the health and wellbeing of older people with multiple needs by preventing deterioration in existing conditions, and providing active support to help them to maintain and regain as much autonomy, independence and resilience as possible, and to make optimal use of individual resources [37].
**Safety**	Prevent adverse outcomes of care (e.g. drug related problems, unnecessary hospitalisations and admissions in long-term care institutions), decrease preventable decline in health status (e.g. falls) and address treatment adherence [38].
**Efficiency**	Affordability of interventions and effective use of infrastructure, resources for sustainability (e.g. hours of service and labour allocated to recipients) and equipment and technology (e.g. IT), and the extent to which interventions may be able to shift activity from acute services to primary care services, improve alignment between the care professionals involved and reduce waste in healthcare spending (e.g. unnecessary readmissions within 30 days) [39,40].

The fourteen initiatives selected according to these criteria show great diversity in the type of care services provided (see Table 2). Their focus ranges from proactive primary care for frail older people and care for older people being discharged from hospital, to nursing care for frail older people, care for people with dementia, and palliative care. More detailed information about the initiatives can be found in an EU report that we wrote about the fourteen initiatives [41].

**Table 2:** Characteristics of fourteen integrated care initiatives participating in the SUSTAIN project.

Country	Region	Integrated care initiative	Type of care services

**Austria**	Vienna	Gerontopsychiatric Centre	Dementia care
	Styria	Coordinated Palliative Care	Palliative care
**Estonia**	Ida-Viru	Alutaguse Care Centre	Home nursing and rehabilitative care
	Tallinn	Medendi	Home nursing
**Germany**	Uckermark	KV RegioMed Zentrum Templin	Rehabilitative care
	Berlin Marzahn-Hellersdorf	Careworks Berlin	Home nursing and rehabilitative care
**Norway**	Surnadal	Surnadal Holistic Patient Care at Home	Home nursing and rehabilitative care
	Søndre Nordstrand in Oslo	Søndre Nordstrand Everyday Mastery Team	Rehabilitative care and mastery of activities of daily living
**Spain**	Osona	Severe Chronic Patients/Advanced chronic disease/Geriatrics Osona	Proactive primary and intermediate care
	Sabadell	Social and health care integration Sabadell	Proactive primary care
**The Netherlands**	West-Friesland	Geriatric Care Model	Proactive primary care
	Walcheren	Walcheren Integrated Care Model	Proactive primary care
**United Kingdom**	Kent	Over 75 Service	Proactive primary care
	Kent	Swale Home First	Hospital discharge planning

### Procedures and measures

**Figure 1:**
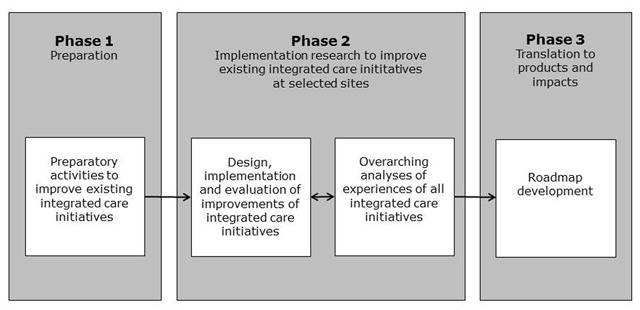
Three interrelated project phases of the SUSTAIN project.

The project is divided into three interrelated phases, namely: the preparation phase (phase 1), the implementation and evaluation phase (phase 2), and the knowledge translation phase (phase 3) (see Figure 1). At the time of writing, the project is in phase 2.

#### Phase 1: Preparation phase

Between autumn 2015 and winter 2016, SUSTAIN research partners established working relationships with the fourteen participating initiatives, and identified relevant local stakeholders related to the initiative (i.e. managers, health and social care professionals, representatives of older people and informal carers, local policy officers). Furthermore, they carried out baseline assessments of each initiative’s principal characteristics and also worked with local stakeholders to identify areas of current practice in the initiative, which might be subject to improvement (e.g. collaboration between formal and informal care providers, involvement of older people in care processes). For the purpose of the baseline assessments, interviews were conducted using a semi-structured interview guide, covering the context and characteristics of the site, stakeholders’ interpretation of SUSTAIN’s four key domains (i.e. person-centredness, prevention-orientation, safety and efficiency) and each site’s performance in relation to them, facilitators and barriers to integration, and potential areas for improvement. At each site, interviews were conducted with the following four study participants: one older person receiving care services from the initiative (user), one informal caregiver, one health or social care professional involved in the initiative and one manager of the initiative. All SUSTAIN research partners used the same interview schedules for their interviews. The interviews were audiotaped with interviewees’ permission.

Interview transcripts from each site were thematically analysed using a uniform, structured template of analysis, generated by structured discussion among research partners. Interview findings were used as inputs for workshops with key stakeholders related to the initiative (i.e. managers, health and social care professionals, representatives of older people and informal carers, local policy officers) at each site. The purpose of the workshops was to discuss outcomes of the baseline assessments and enable sites to determine local improvement priorities.

#### Phase 2: Implementation research to improve existing integrated care initiatives at selected sites

Based on the outcomes of phase 1, local steering groups were set up in spring 2016. Steering groups consists of stakeholders who participated in the workshops together with additional local stakeholders considered relevant to the initiative. These steering groups have been designated to design and implement improvement plans, that is, sets of improvements that apply to local, site-specific priorities and address SUSTAIN’s key domains (e.g. offering training to staff to promote older people’s involvement in care planning and decision-making). Each steering group has agreed to implement their plans over the 18-month period from autumn 2016 to spring 2018.

**Figure 2:**
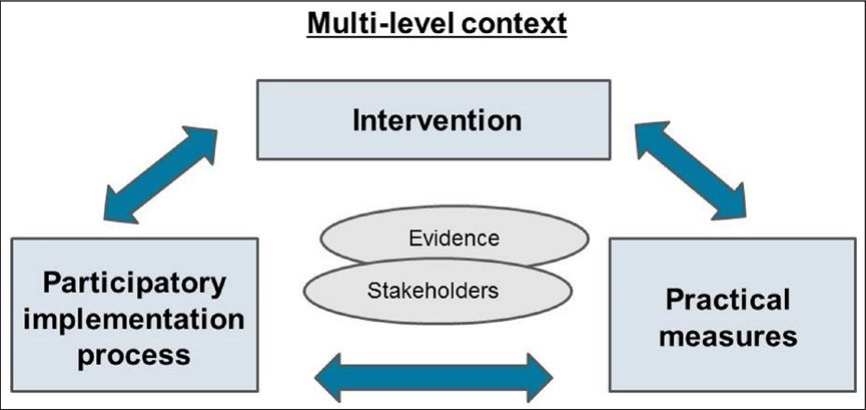
The Evidence Integration Triangle model (adapted) [42].

The implementation of improvement plans and the evaluation of implementation progress and outcomes per initiative are guided by the Evidence Integration Triangle model (see Figure 2) [42]. The Evidence Integration Triangle model, which has its origins in implementation science, supports the effective implementation of theoretical models and scientific evidence in daily practice by tailoring *evidence* to the *multi-level context* (i.e. the historical, political, economic, social, environmental, and cultural settings in which a service/programme is being implemented) in which *local stakeholders* (e.g. managers, health and social care professionals, representatives of older people and informal carers, local policy officers) operate. The Evidence Integration Triangle model thus corresponds well with the objectives of the SUSTAIN project. There are three main components to the Evidence Integration Triangle model:

1. The intervention, which in this case are the improvement plans of the different sites to work towards more person-centred, prevention-oriented, safe and efficient care. The plans include specific areas for improvement (e.g. information exchange between health and social care professionals, assessment of older people’s needs, involvement of older people in the care process) together with actions required to realise such improvements (e.g. creating a shared platform for data sharing, designing a common multidimensional needs assessment tool, training of staff to empower older people). Each site designs and implements its own improvement plan and, consequently, interventions differ across sites;
2. The participatory implementation process, which is the collaboration of SUSTAIN research partners with local stakeholders. Regular meetings between SUSTAIN research partners and steering groups of local stakeholders will take place to design and implement the improvement plans. The research partners will also continue to provide support to the local steering groups by contributing improvement support from theoretical models, scientific evidence and best practices. Following the principles of the Evidence Integration Triangle model, SUSTAIN research partners will further conduct mid-course site-specific analyses so as to share emerging interim site-specific feedback to the steering group about outcomes and progress during the implementation process. In addition, overarching analyses will be undertaken, first, around month 12 of the improvement plans’ implementation. And second, shortly after the end of the 18-month implementation period to compare outcomes and progress at each site and communicate emerging themes to all of them. This will support local stakeholders to further refine their improvement projects and, thereby, foster rapid learning cycles at and between sites [42];
3. Practical measures, a set of qualitative and quantitative data collection tools (see Table 3), for evaluating how the sets of improvements for each of the fourteen initiatives have impacted on SUSTAIN’s key domains. The tools will further allow us to evaluate the implementation progress by focussing on perceptions and experiences of professionals, managers and the steering group of the fourteen initiatives, and on progress in implementing the different components of the improvement plans, including factors that were perceived to facilitate and impede progress. Data will be collected at agreed and specified times during the 18-month implementation period, using the same procedures and tools for all initiatives. In addition to a core set of data collection tools applied in all initiatives, sites are being encouraged to select site-specific tools tailored to their site-specific context and improvement priorities. All data collection tools developed by SUSTAIN research partners are prepared in English and are then translated into the sites’ national languages. Regular meetings and teleconferences take place between research partners to standardise methods of data collection in each country.

**Table 3:** Practical measures for monitoring outcomes and progress of the implementation of the improvement plans.

Item	Data collection tool	Short description

**DEMOGRAPHIC INFORMATION**		
Socio-demographics of older people (users)	Demographic data sheet – older people, administered to older people	Survey developed by SUSTAIN researchers requesting information on age, gender, education, marital status, living situation and self-reported medical conditions
Socio-demographics of informal carers	Demographic data sheet – carers, administered to informal carers	Survey developed by SUSTAIN researchers requesting information on age, gender, education, marital status, relationship and distance to older person (user), paid work and caregiving activities
Socio-demographics of professionals	Demographic data sheet – professionals, administered to professionals	Survey developed by SUSTAIN researchers requesting information on age, gender, nationality and occupation
Socio-demographics of managers	Demographic data sheet – managers, administered to managers	Survey developed by SUSTAIN researchers requesting information on age, gender, nationality and occupation
**OUTCOMES**		
*Person-centredness*		
Patient perceptions of quality and coordination of care and support	The Person Centred Coordinated Care Experience Questionnaire (P3CEQ) [43], administered to older people	Survey measuring older people’s experience and understanding of the care and support they have received from health and social care services
Proportion of older people with a needs assessment	Care plan template (in case sites do not work with care plans, information will be retrieved from clinical notes or other documentation)	Template developed by SUSTAIN researchers for predetermined content analysis of care plans of older people
Proportion of care plans actioned (i.e. defined activities in care plan actually implemented)		
Proportion of care plans shared across different professionals and/or organisations		
Proportion of informal carers with a needs assessment and/or care plan		
Perception and experiences of older people, informal carers, professionals and managers with person-centredness	Semi-structured interviews and focus group interviews with older people, informal carers, professionals and managers	Interview and focus group schedules developed by SUSTAIN researchers including interview items on perception and experiences with receiving person-centred care
*Prevention orientation*		
Perceived control in care and support of older people	Perceived Control in Health Care (PCHC) [37], administered to older people	Survey addressing older people’s perceived own abilities to organise professional care and to take care of themselves in their own homes, and perceived support from the social network
Proportion of older people receiving a medication review	Care plan template (in case sites do not work with care plans, information will be retrieved from clinical notes or other documentation)	Template developed by SUSTAIN researchers for predetermined content analysis of care plans of older people
Proportion of older people receiving advice on medication adherence		
Proportion of older people receiving advice on self-management and maintaining independence		
Perception and experiences of older people, informal carers, professionals and managers with prevention	Semi-structured interviews and focus group interviews with older people, informal carers, professionals and managers	Interview and focus group schedules developed by SUSTAIN researchers including interview items on perception and experiences with receiving prevention-oriented care
*Safety*		
Proportion of older people receiving safety advice	Care plan template (in case sites do not work with care plans, information will be retrieved from clinical notes or other documentation)	Template developed by SUSTAIN researchers for predetermined content analysis of care plans of older people
Proportion of older people with falls recorded in the care plan		
Perception of older people, informal carers, professionals and managers with safety	Semi-structured interviews and focus group interviews with older people, informal carers, professionals and managers	Interview and focus group schedules developed by SUSTAIN researchers including interview items on perception and experiences with receiving safe care, and safety consciousness
*Efficiency*		
Number of emergency hospital admissions of older people	Care plan template (in case sites do not work with care plans, information will be retrieved from clinical notes or other documentation); template to register staff hours and costs	Template developed by SUSTAIN researchers for predetermined content analysis of care plans of older people; template developed by SUSTAIN researchers to collect data on costs and the number of staff hours from local services, organisations or registries
Length of stay per emergency admission of older people		
Number of hospital readmissions of older people		
Number of staff hours dedicated to improvement project		
Costs related to equipment and technology for improvement project		
Perception of older people, informal carers, professionals and managers with efficiency	Semi-structured interviews and focus group interviews with older people, informal carers, professionals and managers	Interview and focus group schedules developed by SUSTAIN researchers including interview items on perception and experiences with receiving efficient care, and finances
**IMPLEMENTATION PROGRESS**		
Team coherence of improvement team (professionals)	Team Climate Inventory – short version (TCI-14) [44,45], administered to professionals	Survey measuring vision, participative safety, task orientation and experienced support for innovation of the improvement team
Perception and experiences of professionals	Focus group interviews with professionals and minutes from steering group meetings	Focus group schedule developed by SUSTAIN researchers including interview items on experienced factors facilitating and impeding outcomes and implementation progress
		Minutes cover progress, issues and contextual issues impacting on outcomes and implementation progress
Perception and experiences of managers	Semi-structured interviews with managers and minutes from steering group meetings	Interview schedule developed by SUSTAIN researchers including interview items on experienced factors facilitating and impeding outcomes and implementation progress
		Minutes cover progress, issues and contextual issues impacting on outcomes and implementation progress

More detailed information about how the Evidence Integration Triangle model is applied in the SUSTAIN project will be described elsewhere [paper in preparation].

*Data analysis*: Data are centrally managed in a secure online database, which is accessible to SUSTAIN research partners. Strict guidelines for data entry have been developed and are shared across research partners. For each site, quantitative and qualitative data analyses will take place to monitor outcomes and progress at the different integrated care sites. Qualitative data will be analysed thematically, quantitative data will be analysed using statistical methods. The coupling of qualitative with quantitative elements is the approach of choice for evaluating complex community-based interventions which are context bound and noted for their differences in application and implementation [14,46,47]. Uniform templates for analysis of each data source are generated through a discussion among research partners. As per the Evidence Integration Triangle model, research partners will conduct site-specific analyses during (month 12; autumn 2017) and at the end (month 18; spring 2018) of the 18-month implementation period to give the local steering groups insight into their progress, and provide them with starting-points for follow up action. In addition, overarching analyses will be undertaken around month 12 and shortly after the end of the 18-month implementation period, in which data from the different sites will be compared and integrated to identify recurring patterns in the implementation of the tailored sets of improvements. In order to enable comparison, uniform procedures for data analysis are being developed. In the overarching analyses, we will follow the principles of the case study design [32]. There will be three steps in our analyses: 1. all data sources will be analysed separately; 2. data will be reduced to a series of thematic statements for each data source; and 3. these site analyses will then undergo a process of pattern-matching across the data from all sites using the identified actions in the improvement plans and SUSTAIN’s propositions (research questions) that have been formulated a priori: I. which actions in the improvement plans are able to improve person-centredness, prevention-orientation, safety and efficiency of care?; II. which actions work for whom, in what context?; III. what are possible explanations for (not) succeeding in improving integrated care?; and IV. what is necessary to guarantee transferability and applicability of actions across the EU for improvement? By comparing outcomes, barriers, facilitators, and experiences, as well as taking into account the characteristics of the study participants and initiatives, we hope to be able to explore how outcomes and implementation progress at the different sites relate to their particular contextual factors and characteristics. In addition to the results of the site-specific analyses, those of the overarching analyses will also be communicated to the different sites so that they can learn from the experiences at the other sites and, where potentially relevant, apply lessons learned to their own context. Comparing and integrating data from the different sites will also support us to ascertain what works for whom, in what context and with what outcomes. As such, we intend to generate EU evidence on improvements to integrated care and their adoption and application to other European health and social care systems, and regions.

#### Phase 3: Translation to products and impacts

SUSTAIN dissemination partners will merge and translate all knowledge and experiences obtained in SUSTAIN to different products for policy-makers and decision-makers from different types of organisations tasked with designing, establishing and maintaining systems of integrated care that focus on older people with complex needs (e.g. national or regional governments, care delivery organisations, and representative organisations of older people and informal carers), during and after the end of the implementation period (spring 2018). This will include the development of an online roadmap, which is a set of instructions, guidelines and proposed actions that provides a step-by-step guide for improving integrated care. In addition, a toolbox will be developed which will be embedded within the roadmap, consisting of a collection of tools (e.g. tool to evaluate person-centredness, tool to evaluate experiences of professionals and managers), lessons learned (e.g. potential solutions for certain implementation issues), scientific evidence and good practices (e.g. actions that will enhance person-centredness), to support the process of improving integrated care. The rationale for developing a roadmap and toolbox is to support the flow of theory, evidence and experiences obtained and observed during the SUSTAIN project into practice. As such, the roadmap and toolbox aim to facilitate the implementation of the tailored sets of improvements at the fourteen sites during the SUSTAIN project, and to facilitate improvements to the way of working for other integrated care sites in Europe after the SUSTAIN project.

### Ethics statement

Ethical approval has been granted by the ethical review committees of Estonia, Spain and the United Kingdom. In Austria, Germany, Norway and the Netherlands, research activities were exempt from the need for ethics committee review as allowed under national standards and regulations. Prior to data collection, informed consent will be obtained for all study participants.

## Discussion

This paper has outlined the overall structure and approach of the SUSTAIN project; a European project designed to improve the current ways of working in fourteen existing integrated care initiatives for older people living at home with multiple health and social care needs.

Many integrated care initiatives for older people with multiple health and social care needs have been introduced in European health systems in a diversity of contexts. These initiatives offer a rich and varied field of practical examples, as described for example in European-wide initiatives including INTERLINKS, the ICARE4EU project and the European Innovation Partnership on Active and Healthy Ageing (EIP-AHA) [48,49,50]. However, there is still discussion on how to measure and evaluate integrated care. This leads to practices being evaluated in different ways, which in turn complicates international comparisons. In SUSTAIN we are not evaluating integrated care programmes as a whole as previous studies have done. Rather we focus on how to identify and implement specific practical improvements to established initiatives based on previous evidence and their experiences with implementation of integrated care to date. We believe SUSTAIN can make important contributions to the research field, especially by contributing to European-wide sharing of evidence and improvement methods by employing uniform procedures for data collection and analysis. As such, we will be able to compare different initiatives across different settings through overarching analyses, thereby encouraging an understanding of generic and contextual factors affecting outcomes and progress of implementation.

In addition to responding to methodological challenges in the area of evaluating integrated care, SUSTAIN further aims to respond to challenges related to knowledge transfer. The translation of project findings and their application into daily practice is fraught with conceptual and operational difficulties. Firstly, scientific evidence is usually developed in isolation from daily practice. As a result, it often fits uncomfortably in the settings and populations where it is intended to be applied, and as such sustainable implementation is scarce. The SUSTAIN project aims to respond to this challenge by taking an action-oriented approach based on the Evidence Integration Triangle model [42], in which local stakeholders and research partners co-design and implement transformative changes. By so doing, the changes implemented are expected to be better tailored to the stakeholders’ context, which in turn may reinforce their motivation to improve current practice and establish sustainable change. Secondly, the rolling-out of the research findings to other health and social care systems, and regions is often limited due to difficulties in translating context-specific knowledge and experience to more generalisable recommendations. As a methodological framework, the Evidence Integration Triangle model provides a consistent approach that, when applied across multiple contexts, may be able to produce generalisable results to make improvements applicable and adaptable to other health and social care systems, and regions.

Besides the opportunities offered by the SUSTAIN project, it should also be noted that our approach poses a challenge to SUSTAIN research partners. Indeed, they will have a dual role during the design and implementation of the tailored sets of improvements in the integrated care initiatives. On the one hand, they are scientific researchers monitoring and evaluating outcomes and progress at the sites. On the other hand, they are facilitators collaborating with local stakeholders by organising meetings, bringing local stakeholders together, and supporting the design and implementation of plans to improve current services. It will be important for SUSTAIN research partners to clearly adjust to these roles and to avoid inappropriately influencing decisions taken by local stakeholders. Firstly because the improvement projects should reflect the preferences of local stakeholders at the sites to encourage the success and sustainability of the improvement projects; and secondly, because lessons learned should be applicable for other contexts not involved in the SUSTAIN project.

A number of other projects aside from SUSTAIN aim to provide guidance to a broader implementation and scaling up of good practices in integrated care across European regions, including SELFIE, ACT@Scale and JA-CHRODIS [51,52,53]. Although each project has its own unique approach, perspective and/or target group, it will be important for these projects to look for opportunities in achieving synergy. Combining each research project’s strengths and perspectives may result in for instance a more comprehensive evidence base or greater consensus on how to evaluate integrated care, upon which we will be able to provide meaningful recommendations to policy-makers and decision-makers and share what can be learned from these European-wide projects.

In conclusion, the SUSTAIN project intends to generate valuable evidence on improving integrated care for older people, and to transfer this knowledge within the SUSTAIN programme and to other regions and health and social care systems in Europe and beyond. By translating lessons learned to products targeted at policy-makers and decision-makers from different types of organisations, we aim to inform and support those managing and improving care for older people with multiple health and care needs.

## Author Information

This paper was published on behalf of the SUSTAIN consortium. People that are or were member of the SUSTAIN consortium when this manuscript was written, are:

Research partners: Maria Marta Arcas Ferre, Eliva Atieno Ambugo, Caroline Baan, Jenny Billings, Gabriela Barbaglia, Simone de Bruin, Carme Carrion, Laia Domingo, Reelika Ermel, Mireia Espallargues Carreras, Erica Gadsby, Christina Häusler, Terje Hagen, Henrik Hoffmann, Kathrine Hofgaard Vaage, Daniëlle Jansen, Kai Leichsenring, Manon Lette, Julie MacInnes, Lina Masana, Eneli Mikko, Mirella Minkman, Peter Müller, Henk Nies, Ulla-Karin Nurm, Giel Nijpels, Konrad Obermann, Gerli Paat-Ahi, Jillian Reynolds, Mari Rull, Georg Ruppe, Sophia Schlette, Riina Sikkut, Monique Spierenburg, Annerieke Stoop, Esther de Weger, Paulina Wosko and Nick Zonneveld.

Knowledge brokering and transfer partners: Borja Arrue, Jeni Bremner, Michele Calabro’, Marie Curran, Sandra Degelsegger, Nick Goodwin, Anne Hendry, Mudi Kadu, Usman Khan, Maggie Langins, Henriikka Laurola, Maude Luherne, Fiona Lyne, Federica Margheri, Nhu Tram, David Smith, Viktoria Stein and Gerald Wistow.
